# The Effects of Physical Activity in Children and Adolescents with Developmental Coordination Disorder

**DOI:** 10.3390/neurolint15030051

**Published:** 2023-06-29

**Authors:** Harilaos Zaragas, Olga Fragkomichelaki, Marina Geitona, Maria Sofologi, Georgia Papantoniou, Dimitrios Sarris, Vassiliki Pliogou, Christos Charmpatsis, Panagoula Papadimitropoulou

**Affiliations:** 1 Department of Early Childhood Education, School of Education, Ioannina Campus, University of Ioannina, 45110 Ioannina, Greece; ppr00184@uoi.gr (O.F.); ppr00159@uoi.gr (M.G.); m.sofologi@uoi.gr (M.S.); gpapanto@uoi.gr (G.P.); dsarris@uoi.gr (D.S.);; 2 Department of Early Childhood Education, School of Education, Florina Campus, University of Western Macedonia, 53100 Florina, Greece; vpliogou@uowm.gr; 3 Department of Educational Sciences and Early Childhood Education, School of Humanities and Social Sciences, Rio Campus, University of Patras, 26504 Rio, Greece; ppapadim@upatras.gr

**Keywords:** developmental coordination disorders, physical activities, sport, comorbidity

## Abstract

The purpose of this literature review was to detect and study the effectiveness of therapeutic intervention programs, such as physical activities and sports, on children and adolescents with Developmental Motor Coordination Disorder (DCD) to improve their motor skills. The sample for this study consisted of 48 (100%) papers, specifically, 40 (83.5%) articles, 3 (6.2%) doctoral theses, 2 (4.1%) master’s theses and 3 (6.2%) papers from conference proceedings from the year 2014 to 2022. To search the sample, the following terms were used: DCD or dyspraxia, physical activity programs, intervention, physical intervention, physical education, etc. The results for the existence of statistically significant results and internal validity of intervention programs using physical activities and sports in children and adolescents with DCD showed that a large number of intervention programs improved the children’s motor skills as well as their daily functionality. In contrast, other interventions failed to improve dynamic and static balance. The negative result could be due either to the short duration of the interventions or to the improper suboptimal design—organization of the methodology of these programs—such as the heterogeneous intervention samples and the use of inappropriate and reliable assessment tools.

## 1. Introduction

Neurodevelopmental disorders include Autism Spectrum Disorder (ASD), Attention Deficit Hyperactivity Disorder (ADHD), Developmental Motor Coordination Disorder (DCD) [1], Mental Disability [2] and Dyslexia [3]. The DSM–5, according to the American Psychiatric Association, states that DCD is a motor skills disorder characterized by significant impairment in the development of motor coordination, with performance in motor activities such as walking and writing being far below expected for the child’s age [4]. A significant decline in academic performance or daily activities is also observed. However, these difficulties affecting movement are not due to the existence of an intellectual disability or neurological disorder (e.g., cerebral palsy) [4]. Ke et al. (2020) [5] reported that being boys, a higher BMI, preterm birth, and certain prenatal conditions are significant risk factors for DCD. When monitoring potential DCD cases, family environmental factors, particularly parenting strategy, should also be considered [5]. DCD usually coexists with the aforementioned neurodevelopmental disorders, which are due to nontypical brain development [6]. Research reports that a number of interventions have been implemented to improve the physical condition of people with DCD [2,7]. In relation to DCD, interventions with physical activities in children and adolescents vary, and each of them focuses on the improvement in different components of DCD, both physically and mentally [8,9]. In addition, positive consequences are observed in cognitive abilities, in social–interpersonal relationships, in the improvement in the emotional state and in the participation of individuals in sports activities in their free time [10].

The implementation of interventions in the various age groups, i.e., children as well as adolescents, leads to the improvement in their motor skills (e.g., locomotion: walking, running, handling skills, dynamic and static balance), motor coordination, gross and fine mobility and in their ambidextrous object handling and orientation in space [11,12,13]. A variety of body exercise methods have been included, such as team training, dual-task training, game-based training and therapeutic riding, trampoline, treadmill, balance beam and swimming [11,14,15].

From all of the above, it appears that there are research gaps both in the international and the Greek scientific field in relation to the use of physical activities and sports as a means of therapeutic intervention in children and adolescents with DCD. The large numbers of therapeutic interventions were mainly of short duration and with a small number of samples or suboptimal design, which leads to statistically nonsignificant results, no generalizability of results and a lack of internal validity [16,17].

## 2. Purpose, Assumptions and Conditions of the Research

The purpose of this literature review was to study the effectiveness of therapeutic intervention programs whose content is physical activities and sports for children and adolescents with Developmental Motor Coordination Disorder (DCD) so that their motor skills can be improved.

Individual objectives of the research were to capture the effect of planned physical activities and sports on the motor and social skills of children and adolescents.

**H1:**  Intervention programs using physical activity are effective in dealing with motor coordination difficulties in preschool children and adolescents.

**H2:**  The intervention programs concerning physical activities and sports are deemed effective for dealing with difficulties in children and adolescents with motor coordination difficulties and accompanying diseases (ADHD, Down’s syndrome, deaf children).

The conditions of the research were to deal exclusively with (a) interventional physical activity and not with programs involving electronic, interactive games or psychotherapeutic sessions, (b) DCD detection tests, (c) physical activity programs for other neurodevelopmental disorders without reports on the DCD and (d) children and adolescents and not for adults over 18 years of age.

## 3. Research Methodology

**Figure 1:**
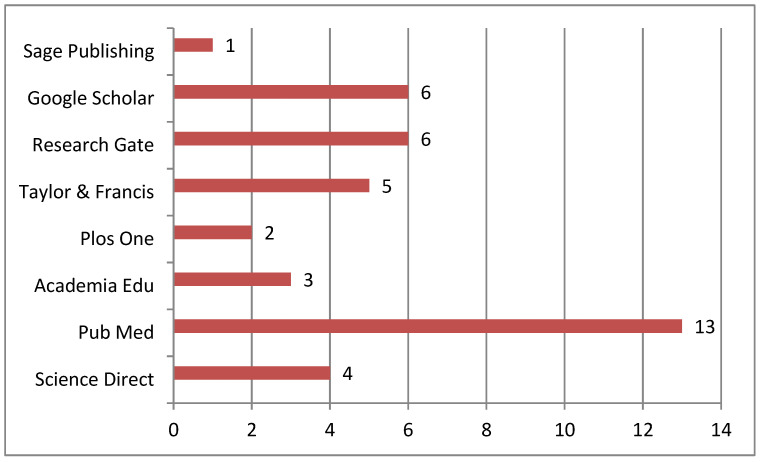
Information sources.

**Figure 2:**
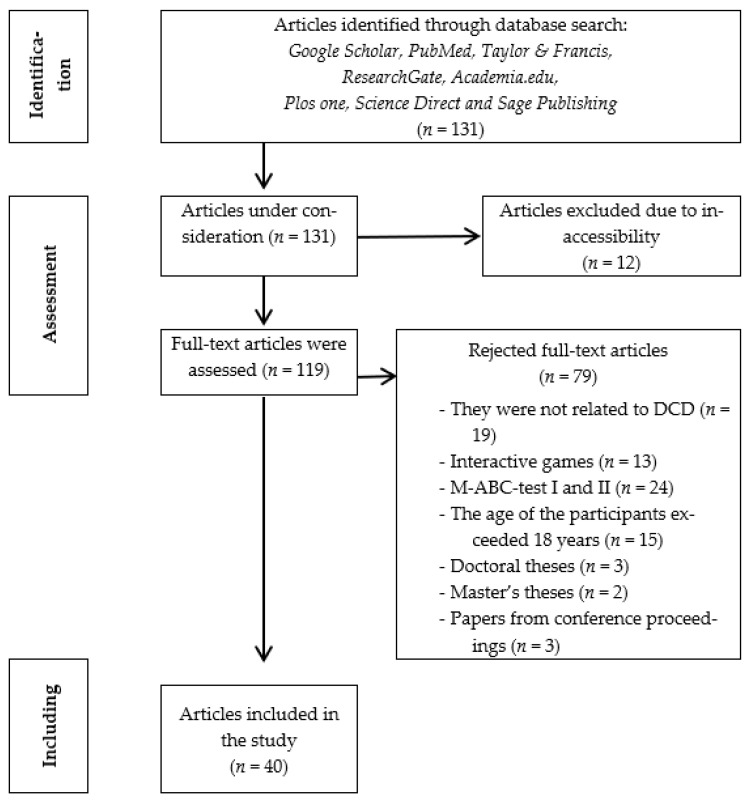
Τhe process of selecting the sample.

This literature review focused on the search and study of physical activity and sports interventions in children and adolescents with DCD. The sample of this study is recommended by articles from reputable scientific journals from the year 2014 to 2022. This systematic review followed the guidelines reported in the Preferred Reporting Items for Systematic Reviews and Meta-Analyses (PRISMA) [18]. The final research sample consisted of targeted studies, which were selected as they answered the research questions and hypotheses. This sample consisted of 40 (100%) articles (see Table 1, Figure 1 and Figure 2).

### 3.1. Eligibility Criteria

Eligibility criteria were designed using the PPAIDFCO design scheme (Participants, Physical Activity Intervention, Duration, Frequency, Comorbidity and Outcomes). Interventions in children and adolescents aged 4 to 18 years, as well as interventions with sports and physical activities, were included. There were restrictions on the English language and the year of publication. Published studies between the years 2014–2022 were also included. Many writers, as noticed from both the international and Greek literature on the literature review in the frame of research methodology, report the following: a research tip for searching for information is to locate recent articles on your topic [19,20]. Only quantitative and qualitative research designs and full-text studies were included in this study. Excluded studies included those that referred to (a) therapeutic interventions for children with neurodevelopmental disorders (ASD, ADHD, cerebral palsy, mental retardation) and did not refer to DCD, (b) the use of electronic, interactive games, (c) exclusively MABC TEST I and II and (d) to adults over 18 years of age (See Table 1). Doctoral theses, postgraduate theses and conference proceedings were not taken into account in this search.

The search resulted in a sample consisting of 131 research studies (123 scientific articles, 3 doctoral theses, 2 master’s theses and 3 conference proceedings). From the above studies, 12 articles were initially removed due to inaccessibility. Then, they removed (a) 19 articles that were about therapeutic interventions for children with neurodevelopmental disorders (ASD, ADHD, cerebral palsy, mental retardation) and did not refer to DCD, (b) 14 articles that made use of electronic, interactive games, (c) 23 articles that referred exclusively to MABC TEST I and II, (d) 15 articles that involved adult participants in the interventions over 18 years of age and (e) 3 doctoral theses, 2 master’s theses and 3 papers from conference proceedings. A final sample of targeted studies was formed that were selected as they answered the hypotheses of the present study. The final sample consists of 40 papers, namely 40 articles (See Figure 2).

### 3.2. Information Sources

To collect the sample, a search was performed in eight electronic data portals: Google Scholar (*f* = 6, 15%), PubMed (*f* = 13, 32.5%), Research Gate (*f* = 6, 15%), Academia Edu (*f* = 3, 7.5%), Plos one (*f* = 2, 5%), Science Direct (*f* = 4, 10%), Taylor & Francis (*f* = 5, 12.5%) and Sage Publishing (*f* = 1, 2.5%) (See Figure 1).

Fourteen (100%) were the countries of origin (*f* = 40, 100%) of the research papers studied in the present literature review. Specifically, they were the United Kingdom (*f* = 2, 5%), Ireland (*f* = 1, 2.5%), Netherlands (*f* = 3, 7.5%), Spain (*f* = 2, 5%), Greece (*f* = 2, 5%), USA (*f* = 2, 5%), Canada (*f* = 3, 7.5%), Australia (*f* = 1, 2.5%), India (*f* = 1, 2.5%), Iran (*f* = 10, 25%), China (*f* = 7, 17.5%), Uzbekistan (*f* = 2, 5%), South Africa (*f* = 2, 5%) and Tunisia (*f* = 2, 5%).

Regarding the years of publication of the articles of the present study (*f* = 40, 100%), we see that from the year 2014 to the year 2021, there is an increasing trend of related publications on interventions through physical activities and sports to restore motor coordination difficulties. According to the present review, they were published in the years 2014 (*f* = 2, 5%), 2015 (*f* = 3, 7.5%), 2016 (*f* = 8, 20%), 2017 (*f* = 2, 5%), 2018 (*f* = 5, 12.5%), 2019 (*f* = 6, 15%), 2020 (*f* = 5, 12.5%), 2021 (*f* = 8, 20%) and 2022 (*f* = 1, 2.5%).

### 3.3. Search Strategy and Study Selection

The search strategy was performed independently by three reviewers and included the following terms and keywords: “DCD” AND “Physical Education” OR “DCD” AND “Sport Activity” OR “DCD” AND “Martial Arts” OR “DCD” AND “Physical Activity” OR ‘Dyspraxia’ AND ‘Body Exercise’ OR ‘DCD’ AND ‘Comorbidity’. A secondary search was performed by reading the reference lists of articles meeting the inclusion criteria for potential additional studies relevant to this review. Full-text transcripts were then independently assessed by two reviewers, and any disagreement regarding the eligibility of included studies was resolved by consensus. Studies that did not meet the inclusion criteria in this second selection phase were excluded.

## 4. Results of the Survey

### 4.1. Results According to the First Hypothesis

#### 4.1.1. Outcomes Related to Preschool Age

De Milander, Coetzee and Vente (2015) examined the implementation of a sensory–motor physical education program in children aged 5 to 8 years (duration 10 weeks), which included sensory–motor coordination, bilaterality and dynamic balance exercises [21]. This intervention led to the improvement in sensory–motor coordination and static and dynamic balance but did not improve the skill of handling objects of the participants (See Table 2). The motor skills training program [22] focused on preschool children aged 4–6 years and focused on strengthening dynamic and static balance, locomotion (running, jumping, walking, etc.) and object handling skills (reception–passing, target accuracy, object handling, etc.). The results showed a significant increase in the overall percentage ranking of the MABC-2 test and an improvement in all motor skills, as well as an increase in the quality of the children’s motor coordination (See Table 2). Additionally examined was the implementation of a physical education intervention program in preschool children aged 4 to 6 years (duration 6 weeks), which was based on improving motor skills (dynamic and static balance, object manipulation and locomotion) [23]. This program led to a significant improvement in the children’s motor skills in the experimental group. The results of the intervention showed that the implemented program was more effective than the standard physical education program applied to preschool children with DCD [23]. Similar positive results to the above research—interventions were those of [24]—who implemented a 10-week motor skills training program (See Table 2). They effectively implemented an intervention program for 5- to 7-year-old boys with adjustment difficulties without a formal diagnosis of DCD [25]. The specific intervention program contained aerobic and muscle-strengthening exercises and lasted 3 weeks. It was a group program with the parents of the children involved in it knowing and encouraging their children’s physical activities (See Table 2).

#### 4.1.2. Outcomes of Combative Contact Sports Interventions

Ma et al. (2018) conducted an intervention in children aged 6–9 years, with Tae Kwon Do exercises, for a total duration of 12 weeks [26]. The results of this intervention showed that an adjusted-intervention TKD can be effective in improving eye–hand coordination in children with DCD (See Table 2).

#### 4.1.3. Interventions Results of Mixed Methods

Many of the interventions studied in the present literature promotion were interventions of mixed methods. These made use of different body exercises, sports activities (exercises with the use of a ball, balance, gross–fine mobility, bicycle, treadmill, table tennis, etc.) and physiotherapy with the aim of the whole some sensory–motor development of children and adolescents with DCD. From these interventions of mixed methods, there was a significant improvement in the participants’ motor skills and specifically in gross and fine mobility, manipulative skills, visual–motor coordination, static and dynamic balance, reaction time and agility, but there was also an important muscle strengthening effect [27,28,29,30,31,32] (Table 2).

#### 4.1.4. Outcomes of Play-Based Interventions

There were interventions that included a variety of sports activities and physical education exercises in a game format in order to be fun and enjoyable for the participants. These interventions led to a significant improvement in motor functions, functional gait, visual–motor and bilateral coordination, gross mobility, running speed and agility, while they led to a statistically nonsignificant improvement in muscle strength. Regarding the improvement or not of static and dynamic balance with play-based interventions, opinions differ as the majority of interventions reported improvement [33], while one intervention reported a statistically nonsignificant improvement [34] (See Table 2).

#### 4.1.5. Outcomes of Interventions Focused on a Specific Activity

A third category of interventions was the focused interventions, which included physical activity exercises that were task-focused (using a ball for target–reception exercises, jumping over an obstacle, balancing on a treadmill). These interventions led to a significant improvement in specific motor skills, static and dynamic balance performance, gross and fine motor skills, manual dexterity, ball handling skills, improvement in MABC-2 test factors, throwing–receiving skills, motor coordination, the adaptation of steps on a treadmill, pacing and also in maintaining the results in remeasurements. However, there was no significant improvement in the ability to move and avoid obstacles [15,22,35,36,37,38,39] (See Table 2).

#### 4.1.6. Outcomes of Interventions Focused on Aerobic Activity

Interventions with aerobic exercises constituted another category of interventions that were studied in the present study. These interventions included aerobic exercises and sports aimed at improving the participants’ motor skills, endurance and muscle strength (e.g., high-intensity exercises, stationary bike, jumping, climbing). Aerobic interventions resulted in significantly improved leg bone strength, improved endurance and reduced fatigue in adolescents with DCD and increased metabolic rate [8] (See Table 2).

#### 4.1.7. Outcomes of Trampoline Interventions

Researchers [14] used the trampoline for balance training (walking exercises, one-leg jumps, one- or both-leg jumps and landings and open or closed landings with eyes and rotations in different directions, using various types of fitness equipment such as cones, ropes, balls, balloons), and the results showed that balance exercises on the trampoline are effective in improving functional results and improving balance performance and motor coordination in children with DCD (See Table 2).

#### 4.1.8. Outcomes of Therapeutic Riding Interventions

Other researchers [40] used Therapeutic Riding in their research for children and adolescents with DCD. The results showed a significant improvement in basic motor skills, including walking, of the participants (See Table 2).

#### 4.1.9. Outcomes of Interventions with Musical Motor Activities

The intervention research [41,42,43,44] used musical motor—rhythmic and dance exercises—as well as aerobic exercises accompanied by music. These significantly improved basic motor skills (See Table 2).

### 4.2. Results According to the Second Hypothesis

A number of researchers [27,29,32,34,40,45] implemented effective intervention programs with physical activities and sports for children with DCD and neurodevelopmental disorders. It emerged that these programs had led both to an improvement in the motor behavior of the participants, as well as to an improvement in their general physical condition, self-efficacy and increased participation in their daily activities (Table 2).

**Table 2:** Summary table of systematic review results.

Number	Author, Author Team, Year of Publication	Total Duration of Intervention and Weekly Frequency of Physical Activity	Comorbidity	Physical Activity	Outcomes
1.	Sit et al. (2019), [46]	8 weeks, 40 min per week	It was not reported	School-based program of Fundamental Movement Skills (FMS) for motor functions, physical activity and other psychological outcomes. Practice 5 specific FMS skills (running, jumping, catching, kicking, and throwing).	FMS training has been shown to promote long-term physical and psychological health by improving certain factors: (1) Motor skills and object control skills. (2) The time spent by children with DCD in moderate to vigorous physical activity both on weekdays and on weekend days. (3) The enjoyment of their participation in physical activity during free time. These improvements were still evident 12 months after the intervention.
2.	Hession et al. (2019), [40]	2 times on September of 2013 and 8 weeks (January—February 2014), 1 session per week for 30 min	Attention Deficit Hyperactivity Disorder (ADHD), Asperger, Autism Spectrum (ASD) Disorders, Sensory Processing Disorder, Hypotonic Muscle Tone	Two intervention programs: (1) Therapeutic riding, (2) Audiovisual with exposure to the rhythm and movement of horses.	(1) Reduction in problematic behavior in both intervention groups (an improvement over time in emotional and behavioral functioning). (2) Reduction in depressive symptoms in both groups (3) Positive effect on the social response (social functioning) of the children of the two groups (4) Positive effect on gait and gross motor coordination in the therapeutic riding group, while no statistically significant changes were observed in the control group.
3.	Díaz-Pérez et al. (2020), [41]	10 weeks, 1 session per week for 50 min	It was not reported	Music–motor intervention program based on music, movement and dance. Specifically, singing games, (b) non-singing games and (c) dancing games.	Only the children in the intervention group had significant changes, improving their motor performance. This highlights the effectiveness of the program based on dance, movement and music activities as it activates cognitive areas. Indeed, many of the children who participated in the intervention program left their difficulties behind and achieved scores on the MABC-2 so that they were considered outside the risk level of motor coordination problems.
4.	Zwicker et al. (2014), [31]	2 weeks for July 2012 and 2 weeks for July 2013, 4 sessions per 2 weeks for 90 min each time	It was not reported	Intervention program implemented in a summer camp for children with DCD based on (1) Cognitive Orientation to daily Occupational Performance (CO-OP)—Functional Kinetic Objectives, (2) the sports activities that the camp program had (hiking, climbing).	The children themselves perceived a significant improvement in the achievement of their chosen goal and felt satisfied with their performance in relation to these goals. Self-confidence increased as the camp provided children with opportunities for trying new activities, learning strategies, being and working with other children with DCD, and knowledge about DCD. Parents of participants in both camps reported that their children continued to use the strategies learned at camp. No significant improvement was found in children’s perceived self-efficacy, goal-setting, self-concept and competence in predisposition to physical activity before and after the camp.
5.	Heus et al. (2020), [29]	20 sessions, 1 session per week for 60 min each time	ADHD, ASD and persons with possible existence of ADHD, ASD	An intervention program implemented in a rehabilitation center for DCD in which were used, among others, (1) Cognitive Orientation to daily Occupational Performance (CO-OP) (2) Neuromotor Task Training methods.	Positive results: (1) In setting goals to rehabilitate problems in everyday life. (2) In the quality and speed of execution of daily projects only for children between 5 and 8 years old. (3) In MABC-2. In some subscales, there were significant differences; however, these differences were very small regarding (1) the degree to which a child has problems in different types of behavior related to scales of cognitive functioning and (2) the measurement of general aspects of the quality of life, as related to health, was shown.
6	Rahman et al., 2021, [47]	16 sessions, 3 times per week for 45 min	It was not reported	Included in Spark program: warm-up, motor skills, games such as jump rope, walk, shoot the ball, aiming and throwing the ball into the basket and bowling, based on educational activities in the form of a game to investigate its effects on some neuropsychological variables.	The intervention with the Spark program appeared to have a significant effect on some motor and neuropsychological performances of children with DCD. Significant improvement in manual dexterity, aiming and grasping and balance. Minor change to the Threading Lace component.
7.	Cheng et al., 2019, [48]	3 months, 2 sessions per week for 40 min each time	ADHD, ASD and Dyslexia	Neuromuscular training (NMT) program with exercises focused on adaptive balance performance and leg muscle activation.	Short-term neuromuscular training (NMT) failed to improve adaptive balance performance and leg muscle activation times in children with DCD.
8.	Yam et al., 2019 [49]		ADHD, ASD	The use of Kinesio Tape (KT) on dynamic balance of the trunk and related muscle activity of the lower limbs in children with DCD.	Kinesio Tape (KT): (1) It had a direct beneficial effect on dynamic balance performance. (2) Positive results in the control of neuromuscular balance. (3) Increase in leg muscle peak activation and time-to-peak muscle activation of the dominant lower limb.
9.	(A) Fong et al., 2016 [36]	3 months, 2 times per week for 90 min each time	ADHD, Dyslexia and persons with possibility of existence, ASD	Comparison of two intervention programs: (1) FMPT (Functional Movement Power Training) dynamic training exercises were intended to improve the posture of muscle strength and the speed of contraction in the legs. (2) FMT (Functional Movement Training) program (training in a specific task with electromyographic (EMG) biofeedback for rehabilitation motor learning difficulties and enhancing neuroplasticity and balance performance).	The FMPT program was more effective than the conventional FMT program in enhancing neuromuscular performance in children with DCD. FMPT effective intervention to improve balance strategies, peak knee extension strength and time-to-peak knee flexion strength.
10.	De Milander et al. (2015), [21]	10 weeks, 2 times per week, 30 min each time	It was not reported	Sensory–motor program based on Kinder Kinetics. Sensory–motor coordination, bilaterally, dynamic–static balance exercises.	After the intervention, the balance showed a significant change. Manual dexterity and aiming and grasping showed no significant changes. These three components contributed to the overall score, which revealed a nonsignificant difference in overall motor skill levels.
11.	Farhat et al. (2016) [35]	8 weeks, 3 times per week, 60 min each time	It was not reported	Group intervention in a school context-oriented towards the task of training skills in the motor and physical abilities of children with DCD (practice: agility, aerobic power, neuromuscular strength training, flexibility, balance, ball balance, reaction speed, aerobic power, ball skill, strength coordination, ball handling).	The results showed that 10 children in the intervention group with DCD improved their performance on the MABC test. Additionally, the intervention group with DCD children improved significantly in manual dexterity, ball skills and balance. Significant performance improvements were also found in sprinting, lower extremity strength and power, writing quality, overall fine motor skills (in which no intervention was made) and speed. Overall gross motor coordination and physical ability improved. The children in the intervention group also seemed to enjoy the process.
12.	Farhat et al., 2015, [50]	8 weeks, 3 times per week, 60 min	It was not reported	Practice in the following skills: agility, aerobic power, neuromuscular strength training, flexibility, balance, balance ball skill, speed reaction speed, aerobic power-ball skill, strength coordination, ball handling).	The intervention group of children with DCD: (1) Have a delay in anaerobic threshold and an improvement in aerobic endurance and tolerance to physical activity. (2) Have improved walking distance (3) Have a higher maximum heart rate (4) Have reduced perceptual physical fatigue (exertion).
13.	Saidmamatov et al., 2021, [22]	10 weeks, 2 times per week for 45 min	It was not reported	Motor skills training program with exercises in the areas of manual dexterity, throwing–catching and balance.	In general, the quality of motor skills of children in the intervention group with DCD improved. Specifically, there was an improvement in manual dexterity, aiming/throwing, grasping and balance, as well as an improvement in the overall MABC-2 final score in the intervention group. The effectiveness of the intervention was similar for both sexes.
14.	Navarro-Patón et al., 2021, [23]	6 weeks, 1 session per week, for 40 min	It was not reported	Intervention program based on different motor skills (manual dexterity, aiming/throwing, catching and balance).	Postintervention measures showed improvements in manual dexterity, aiming/throwing and catching and balance, as well as the intervention group’s overall final score.
15.	Giagazoglou et al., 2015, [14]	12 weeks, 3 times per week, 45 min each time	It was not reported	Balance training program with balance exercises in circuit training, which also included exercises on the trampoline.	Significant performance improvement in all balance post-tests. Despite the fact that most of the exercises were performed with eyes open, the balance of the participants without visual control was also significantly improved.
16.	Saidmamatov Ozodovich (2021), [24]	10 weeks, 1 time per week, 45 min each time	It was not reported	Motor skills training program (exercises of fine and gross mobility, motor coordination, balance, difficulty of the proprioception, touch, kinesthetic sensation, relaxation).	Improvement in motor skills (relative to the factors of MABC-2 and the control group except for one child).
17.	Dannenbaum et al., 2021, [34]	5 days, 5 h each day	ADHD, language disorder (verbal apraxia, dysphasia), Anxiety Disorder, idiopathic quadriceps contraction, sleep apnea, epilepsy	Vestibular dysfunction rehabilitation camp in a hospital setting (Vestibular Rehabilitation) with rehabilitation exercises in the form of a game (Static–Dynamic balance, trunk muscle strength, gaze stability, coordination, multisensory integration).	The results showed that the intervention produced significant improvements in functional gait and gaze stability. Motor function, static balance and participation outcomes were unchanged. It is interesting to note that while camp counselors subjectively noted improved quality of movement, improved stability, reduced need for guidance/corrections, and enhanced independence in performing various activities, these improvements were not reflected in many measures. The scores in the measurements related to the participation appeared higher, though, after the intervention and in the measurement at the follow-up, but it did not significantly affect the participation of the children in their daily environment. Another positive outcome of the intervention was the new friendships and camaraderie that developed.
18.	Yu et al., 2016, [9]	6 weeks, 2 times per week for 35 min each time	It was not reported	Fundamental Movement Skills (FMS: running, jumping, catching, throwing, kicking) training on FMS proficiency, self-perceived physical ability, physical activity, and sleep disturbance in children with DCD.	Improvement in motor skills (jumping) and object control skills (grasping and kicking) of children with DCD. Their improvements in object control skills (catching and throwing) were maintained for at least 6 weeks. Improvement of self; children’s perceived physical ability with DCD regarding physical coordination, physical strength and fitness immediately after training. Children with DCD showed fewer sleep disturbances 6 weeks after the intervention. Participation in physical activity did not increase.
19.	Kuijpers et al., 2019, [15]	6 sessions, 2 times per week for 30 min each time	It was not reported	Task-oriented treadmill training with projected visual context (C-mill) (step adjustment exercises, obstacle avoidance, hitting, targets).	Significant improvement in complex gait adaptation tasks and maintenance of results after remeasurement 6 months later in treadmill step adaptation exercises. There was also a generalization of the results of the intervention even when there was no treadmill exercise and an improvement in gait, but a nonsignificant improvement in the obstacle course and the single run.
20.	Tan et al., 2020, [8]	13 weeks, 2 sessions for 90 min per week	At risk for osteoporosis	Multimodal exercise intervention for bone health (cardiovascular exercises, core strength and flexibility exercises, motor skills and postural skills, resistance training for the lower body, resistance training in general, Plyometrics, team games and partner games).	Effective intervention in the improvement in muscle and bone parameters (at the tibial site for bone mass, cortical area). Lower body fitness measures were significantly associated with improvements in parameters of bone health. The improvement in bone parameters was maintained for at least 3 months.
21.	Hashemi et al., 2016, [28]	8 weeks (24 sessions), 3 sessions per week for 60 min each time	It was not reported	Set of natural activities (set of physical exercises aimed at strengthening coordination, control, inhibition, response and reaction time).	Significant improvement in balance, bilateral coordination, response speed, visual–motor control, speed and dexterity of the upper limb. No significant changes were observed in the components (1) speed and dexterity and (2) strength.
22	Balayi and Sedaghati, 2021, [27]	8 weeks, 3 sessions per week for 60 min each time	Intellectual Disability	Combination of physiotherapy exercises; hemsball. In motor skills, trunk stability exercises with coordination exercises in motor proficiency.	The intervention group improved in running speed, agility, balance, two-way coordination, strength, upper extremity coordination, response speed, motor–visual control, upper extremity agility and general fine and gross motor skills.
23.	Fong et al., 2016, (2nd), [39]	3 months, 2 sessions per week for 90 min each time	ADHD, Dyslexia, suspected ASD	Task-specific balance training in the form of functional movement in the form of an FMT program (two-legged balance on foam with electromyographic biofeedback, balance with one foot on the ground (alternate leg), walking in a straight line with raised heels, two-legged bounce, balance on a ball while the person is walking.	In general, task-specific balance training was found to marginally improve body–sensory function and somewhat improve the balance performance of children with DCD. Specifically, the FMT group showed greater improvements than the control group in the body–sensory ratio in 3 and 6 months, but within-group changes were not significant. The balance performance of the FMT group was significantly better than that of the control group in 3 and 6 months.
24.	Damanpak and Sabzi, 2022, [33]	8 weeks, 3 sessions per week, 45–60 min each session	It was not reported	Kinetic games to improve executive functions (coordination, balance, fine and gross mobility, perception, sensory–motor exercises).	Improvement in the executive functions of the intervention group, specifically the skills of attention, organization, inhibition, planning and decision-making.
25.	Maharaj and Lallie, 2016, [30]	8 weeks, 30 min per week	It was not reported	Intervention program with an emphasis on gross mobility exercises: trunk stability, strengthening, balance and coordination exercises (target throwing ball exercises with an emphasis on strength and coordination, mimicking sensory integration exercises).	Improvement in gross motor function. Improvement in M-ABC and DCDQ score. Some teachers’ ratings of the children’s motor skills were lower than those of the children’s parents.
26.	Ma et al. (2018), [26]	12 weeks, 1 session each week, 60 min each time and home physical activities 7 times per week	ADHD, ASD, Dyslexia	Taekwondo training intervention for skeletal development and motor performance (balance control and eye–hand coordination training. Mostly kicking and striking techniques were used).	Overall improvement in the skeletal development of the TKD group in the movement time of EHC (eye–hand coordination) in children with DCD. (1) Skeletal growth improved in both groups over time (the TKD group had a significant delay in skeletal growth at baseline compared to the control group). (2) Improvements in MABC scores were also observed in both groups over time. (3) Only the TKD group had a significant improvement in EHC (eye–hand coordination) movement time at 3 and 6 months. Both groups had general improvements in gross and fine motor skills over time (this may also be due to the maturation factor). In terms of static standing balance performance, surprisingly, neither group showed significant improvement over time. For motor performance, the factor of maturation may be more responsible.
27.	Kane and Staples (2014), [25]	7 weeks, 2 h each session, 3 times per week	ADHD, anxiety, in-toeing, low birth weight, premature birth, speech delay, average intellectual disability, delay in language expression, possible autism, apraxia of speech, abnormal brain development, epilepsy.	Group program of gross motor skills with an emphasis on the participation of parents in the performance of their children’s motor skills and physical activity (multidisciplinary program; aerobic and strength exercises and practice skills directly related to each child’s goals).	Positive effects on mobility. Parents’ satisfaction and perception of their child’s performance also improved. The majority of children also reported improved performance, at least on the targets. On average, moderate to vigorous physical activity improved by 10 min per day, although these gains were not significant. Time spent in sedentary activities was unchanged.
28.	Jahanbakhsh et al., 2020, [37]	8 weeks, 3 times per week, 45 min each time	It was not reported	Task-specific balance training in single-task and dual-task conditions for balance performance. (1) Single-task conditions: children had to maintain their balance while standing on one leg and maintain their balance while walking. (2) Dual-task conditions: similar to the single-task group program, with the exception that the children also had to perform cognitive tasks such as counting numbers.	Improvement in both groups’ static and dynamic balance, with the dual-task group having more improvement and the results remaining even two months after the program had been completed. Therefore, a dual-task training program that focuses on balance and cognitive tasks may improve children’s static and dynamic balance skills more than single-task training.
29.	Kordi et al., 2016, [51]	12 weeks, 2 sessions per week, 60 min each session	It was not reported	Program strength exercises for the improvement in static and dynamic balance.	Significant increase in muscle strength of children with DCD and improvement in their static balance performance. Exercises had no significant effect on dynamic balance.
30.	Noordstar et al., 2017, [52]	12 weeks, 1 time per week for 30 min	It was not reported	(1) Motor intervention (ball exercises, basketball), (2) motor intervention (ball exercises, basketball)	No differences were found between the intervention group and the care-as-usual group. The children improved their motor performance and increased their perceived athletic ability, their global self-esteem and perceived motor ability. The improvement was maintained at the repeat measurement 3 months after the intervention.
31.	Caçola et al., 2016, [53]	10 weeks, 1 time per week for 60 min	It was not reported	Group A: Task-oriented training. Group B: Basic motor skills training.	The children improved their motor skills after both programs, but: (1) After Program A, they showed higher anxiety and lower levels of enjoyment, even though parents noted an improvement in functioning rate and a decrease in peer problems. (2) After Program B, children’s anxiety levels decreased and parents noticed better control of their movements.
32.	Mohamma et al., 2018, [44]	8 weeks, 1 time per week	Depression, Anxiety disorder	Aerobic rhythmic training intervention program.	Improvement in motor skills of the intervention group, and a significant reduction in the levels of anxiety and depression of the intervention group. The intervention was fun and enjoyable for the participants of the intervention group.
33.	Tamplain et al., 2020, [32]	10 weeks, 1 time per week for 60 min	ADHD, Dyslexia, Dysgraphia, Obsessive Compulsive Disorder (OCD), Sensory Processing Disorder.	Collaborative motor exercises, motor coordination exercises, gross mobility exercises, fine motor improvement exercises, trunk stabilization exercises, and dynamic balance exercises.	Improvement in dynamic stabilization and trunk control and improvement in static and dynamic balance.
34.	Zolghadr et al., 2019, [45]	12 weeks, 3 times per week	It was not reported	Static and dynamic balance exercises.	Improvement in neuromuscular and bilateral coordination of the intervention group
35.	Rezaei et al., 2016, [38]	8 weeks, 3 times per week for 45 min	It was not reported	Static and dynamic balance exercises.	Significant improvement in the accuracy of bilateral upper extremity coordination in the intervention group and improvement in attention and concentration in the intervention group.
36.	Norouzi et al., 2021, [54]	4 weeks, 2 times per week for 40 min	It was not reported	Bilateral coordination exercises with QET (Quiet Eye Training) or TT (Traditional Training: flexions and extensions in in-phase and antiphase movements of the upper limbs).	Significant improvement in the accuracy of bilateral upper extremity coordination of children in the Quiet Eye intervention group compared to the Traditional Training intervention.
37.	Liu et al., 2018, [38]	3 weekly sessions	Neurological disabilities, motor disability.	Exercises on an ergometric stationary bike.	Improving endurance and reducing fatigue in adolescents with ADHD and increasing metabolic rate. Greater fatigue was observed during sessions in adolescents with DCD, which is useful for creating interventions for people with DCD.
38.	Monastiridi et al., 2021, [55]	12 weeks, 3 times per week For 60 min	It was not reported	Functional training program for core stabilization/strengthening (core, balance, strengthening exercises).	Significant improvements in motor performance, static and dynamic balance, abdominal muscle strength and endurance, and lower back and hamstring flexibility. Improvement in Body Mass Index (BMR), quality of life and functionality in the intervention group.
39.	Wood et al., 2017, [56]	4 weeks, 1 time per week for 60 min	It was not reported	(1) Quiet Eye Training group: focus on a target position before throwing, and observe the ball before serving. (2) Technical Training group: video instructions on throwing and serving phases.	Improving eye control and coordination of throwing and passing the ball in the QET (Quiet Eye Training) Group. Improving self-confidence, social skills and disposition for physical activity in the QET group.
40.	Alagesan et al., 2020, [39]	8 weeks, 3 times per week	Anxiety Disorder	Aerobic training (brisk walking, skipping rope, running, jogging, aerobic dance, hide and seek, walking).	Improving the quality of life, academic results, physical activity and functionality of people with DCD. Reduction in the increased level of anxiety of people with Anxiety Disorder.

## 5. Case Control

### 5.1. First Case Control

The testing of the first hypothesis regarding the results for the existence of statistically significant results and internal validity of intervention programs using physical activities and sports in children and adolescents with DCD showed that a large number of intervention programs improved children’s motor skills, as well as their daily functionality. Conversely, other interventions failed to improve dynamic and static balance [12,48,51,57]. This negative result could be due either to the short duration of the interventions or to the improper suboptimal design—organization of the methodology of these programs—such as the heterogeneous intervention samples and the use of inappropriate and reliable assessment tools [46].

Also, it became clear that in various research, the improvement in the sensory–motor skills of the participants was not due to the implementation of the intervention programs but to other external environmental factors (e.g., in parallel physical therapy sessions) [11]. From the above, it is understood that a large number of interventions have low reliability, risk of bias and, consequently, lack of internal validity [10,12,16,58,59]. Finally, with regard to external validity, various research of the global scientific community was mainly applied to small samples, which may well lead to a lack of generalizability of the results and a lack of external validity [16,57,59]. 

### 5.2. Second Case Control

The verification of the second hypothesis showed that a great amount of research has been conducted for the implementation and investigation of the benefits of physical education and sports intervention programs for improving motor skills, fitness, self-efficacy and quality of life in children and adolescents with DCD and neurodevelopmental disorders. The researchers included in their interventions children and adolescents with Attention Deficit Hyperactivity Disorder, Autism Spectrum Disorder, Sensory Integration-Processing Disorder, Developmental Verbal Dyspraxia, Dyslexia, Sleep Apnea, Dysgraphia and Obsessive Compulsive Disorder. These programs had led both to an improvement in the motor behavior of the participants as well as to an improvement in their general physical condition, self-efficacy and increased participation in their daily activities.

## 6. Conclusions, Discussion

From the present literature review, it has been shown that there are intervention programs that use physical activity in children and adolescents with DCD and are effective in improving their motor status behavior. However, apart from the positive effects on motor skills, it appeared that interventions using physical activities have some other effects on the social and emotional development of children and adolescents with DCD, either positive or negative. Specifically, aerobic exercise has been shown to reduce levels of anxiety and depression [44], as well as improve quality of life and academic performance [39]. In addition, game-based physical education programs have been shown to improve the skills of attention, organization, self-control, planning and decision-making, executive functions and self-efficacy [33].

Gross mobility programs appeared to create feelings of pleasure and enjoyment in participating in the intervention, to improve attention expectation, cooperation, levels of participation in moderate to vigorous physical activity, self-efficacy and, finally, to improve parental perceptions regarding their child’s performance in physical activity [25,30]. In relation to the programs that had trunk stabilization exercises, some quality-of-life health factors of the individuals appeared to improve, as well as their functional status [55]. Regarding Body Mass Index (BMI) and trunk stabilization exercises, research results are ambiguous. Specifically, one study reports a significant improvement in BMI, while the other reports that there were no significant differences in BMI [55]. Additionally, high- and low-intensity physical activity programs that increased metabolic rate reduced adolescent fatigue after the end of each session and increased fatigue during sessions [38].

There were also two interventions implemented in the context of the camp, strengthening self-efficacy, self-confidence, attention, organization and communication skills, as well as participation in extracurricular activities [31,34]. In addition, the program using horse riding improved the social and emotional skills, behavior and cognitive abilities of the participants [40]. After the end of an intervention focusing on fundamental motor skills, children showed fewer sleep disturbances [9]. In a program with task-oriented activities, with motor skills training among children, after the end of the intervention, the children appeared to show higher anxiety and lower levels of enjoyment, even though their parents noted an improvement in rhythm functioning and a reduction in problems with peers [53]. In the same intervention when the children themselves chose the skills they wanted to be trained on, the children’s anxiety appeared to decrease [53]. Quiet Eye interventions have been shown to improve attention and concentration in children with DCD, and, in addition, parents report improvements in their child’s self-confidence, social skills, and disposition for physical activity after the intervention [54,56]. In addition, dual-task balance exercises have been shown to improve cognitive abilities [37]. 

The admissions of the present bibliographic review are that it dealt with published scientific articles (a) in English and not in any other language, (b) of the last nine years from 2014 to 2022, and (c) the study of risk bias and bias assessment reporting was not thoroughly investigated. The advantages of the present literature review are that it presents (a) the results of all investigations and possible causes of heterogeneity between study results and (b) the results of all analyses carried out to assess the robustness of the synthesized results. 

This review has strong points as well as limitations. Screening, data extraction, synthesis and meta-analysis are recorded as strong points by the authors. Deliberate efforts have been made in an open and transparent manner to ensure that the presentation and comparisons of results are meaningful. Some readers may disagree with the way the comparison of these studies was conducted or may think of additional necessary comparisons. Additionally, this process was made more difficult by poor descriptions of the process, with many authors describing the intervention methods in general terms, i.e., the intervention was mentioned as a motor skills training program (fine and gross motor exercises) without the detailed description of the physical exercises.

### 6.1. Teaching Instructions and Educational Applications

At all levels of the educational system, there is a standard program of physical education and sports. From the study of the above papers, it seems that this is neither for all children nor enough to eliminate the difficulties that appear due to the modern way of life. Formal education in Greece from the earliest grade of kindergarten should place more emphasis on Physical and Psychomotor Education Programs. Of course, there is the official detailed Physical Education Curriculum, but despite this, often, the lack of logistical infrastructure and the insufficient training of teachers regarding the weaknesses and deficiencies in motor coordination lead to the conclusion that additional training is needed for kindergarten and physical education teachers for the systematic implementation of DCD treatment programs through targeted physical activities. This emphasis should be given with more appropriate and more attractive physical activities adapted to the developmental needs and interests of the children, but also with the appropriate relevant training of teachers for dealing with DCD.

### 6.2. Future Research

It would be interesting to study the relationship between physical activity programs and sports in combination with the method of mental imagery to eliminate motor coordination difficulties and, at the same time, strengthen motor skills.

## Figures and Tables

**Table 1:** Eligibility criteria used for the study selection and Information sources.

Category	Inclusion Criteria	Exclusion Criteria
Participants	Studies for children and adolescents from 4 to 18 years of age with DCD	Studies with adults over 18 years of age
Physical Activity Intervention Duration Frequency	Studies with sport activities, martial arts, aerobic exercises, swimming, trampoline, etc. studies on interventions for children with DCD but also with neurodevelopmental disorders (ASD, ADHD, cerebral palsy, mental retardation).	Studies on interventions in children with neurodevelopmental disorders (ASD, ADHD, cerebral palsy, mental retardation) and not refer to DCD. Doctoral theses, postgraduate theses and conference proceedings.
Comorbidity		
Outcomes	Assessment positive or negative	Doctoral theses, postgraduate theses and conference proceedings

## Data Availability

Not applicable.

## References

[1] Hession C.E., Law Smith M.J., Watterson D., Oxley N., Murphy B.A. (2019). The impact of equine therapy and an audio-visual approach emphasizing rhythm and beat perception in children with developmental coordination disorder. J. Altern. Complement. Med..

[2] Díaz-Pérez A., Vicente-Nicolás G., Valero-García A.V. (2020). Music, body movement, and dance intervention program for children with developmental coordination disorder. Psychol. Music.

[3] Ludyga S., Pühse U., Gerber M., Kamijo K. (2021). How children with neurodevelopmental disorders can benefit from the neurocognitive effects of exercise. Neurosci. Biobehav. Rev..

[4] Ku B. (2020). The effects of motor skill interventions on motor skills in children with developmental disabilities: A literature review. Asian J. Kinesiol..

[5] Sung M.C., Ku B., Leung W., MacDonald M. (2021). The effect of physical activity interventions on executive function among people with neurodevelopmental disorders: A meta-analysis. J. Autism Dev. Disord..

[6] American Psychiatric Association (2013). Diagnostic and Statistical Manual of Mental Disorders.

[7] Du W., Ke L., Wang Y., Hua J., Duan W., Barnett A.L. (2020). The prenatal, postnatal, neonatal, and family environmental risk factors for Developmental Coordination Disorder: A study with a national representative sample. Res. Dev. Disabil..

[8] Dewey D., Bernier F.P. (2016). The concept of atypical brain development in developmental coordination disorder (DCD)—A New Look. Curr. Dev. Disord. Rep..

[9] Lee D., Psotta R., Vagaja M. (2016). Motor skills interventions in children with developmental coordination disorder: A review study. Eur. J. Adapt. Phys. Act..

[10] Τan J., Siafarikas A., Hands B., McIntyre F., Hart N., Rantalainen T., Chivers P. (2020). Impact of a multimodal exercise program on tibial bone health in adolescents with development coordination disorder: An examination of feasibility and potential efficacy. J. Musculoskelet. Neuronal Interact..

[11] Yu J., Sit C.H., Burnett A., Capio C.M., Ha A.S., Huang W.Y. (2016). Effects of fundamental movement skills training on children with developmental coordination disorder. Adapt. Phys. Act. Q..

[12] Preston N., Magallón S., Hill L.J., Andrews E., Ahern S.M., Mon-Williams M.A. (2017). Systematic review of high quality randomized controlled trials investigating motor skill programmes for children with developmental coordination disorder. Clin. Rehabil..

[13] Engel A.C., Broderick C.R., van Doorn N., Hardy L.L., Parmenter B.J. (2018). Exploring the relationship between fundamental motor skill interventions and physical activity levels in children: A systematic review and meta-analysis. Sport. Med..

[14] Miyahara M., Lagisz M., Nakagawa S., Henderson S.E. (2017). A narrative meta-review of a series of systematic and meta-analytic reviews on the intervention outcome for children with developmental co-ordination disorder. Child Care Health Dev..

[15] Smits-Engelsman B., Vinçon S., Blank R., Quadrado V.H., Polatajko H., Wilson P.H. (2018). Evaluating the evidence for motor-based interventions in developmental coordination disorder: A systematic review and meta-analysis. Res. Dev. Disabil..

[16] Giagazoglou P., Sidiropoulou M., Mitsiou M., Arabatzi F., Kellis E. (2015). Can balance trampoline training promote motor coordination and balance performance in children with developmental coordination disorder?. Res. Dev. Disabil..

[17] Kuijpers R., Smulders E., Groen B., Weerdesteyn V., van der Sanden N. (2019). Task-oriented treadmill training improves gait adaptability in children with Developmental Coordination Disorder. Gait Posture.

[18] Cameron K.L., Albesher R.A., McGinley J.L., Allison K., Cheong J.L.Y., Spittle A.J. (2020). Movement-based interventions for preschool-age children with, or at risk of, motor impairment: A systematic review. Dev. Med. Child Neurol..

[19] Kim M.J., Choi J.S. (2021). A systematic review of developmental coordination disorders in South Korea: Evaluation and intervention. J. Korean Soc. Sens. Integr. Ther..

[20] Page M.J., McKenzie J.E., Bossuyt P.M., Boutron I., Hoffmann T.C., Mulrow C.D., Shamseer L., Tetzlaff J.M., Akl E.A., Brennan S.E. (2021). The PRISMA 2020 statement: An updated guideline for reporting systematic reviews. Syst. Rev..

[21] Cresswell J., Cresswell D. (2019). Research Design Qualitative, Quantitative and Mixed Methods Approaches.

[22] Papanastasiou K. (1996). Methodology of Educational Research.

[23] De Milander M., Coetzee F.F., Venter A. (2015). Perceptual-motor intervention for developmental coordination disorder in Grade 1 children. South Afr. J. Res. Sport Phys. Educ. Recreat..

[24] Saidmamatov O., Raximov Q., Rodrigues P., Vasconcelos O. (2021). A ten-week motor skills training program increases motor competence in children with developmental coordination disorder. Children.

[25] Navarro-Patón R., Martín-Ayala J.L., Martí González M., Hernández A., Mecías-Calvo M. (2021). Effect of a 6-week physical education intervention on motor competence in pre-school children with developmental coordination disorder. J. Clin. Med..

[26] Saidmamatov O., Ozodovich R.Q. (2021). Improving the motor skills of children with developmental coordination disorder. J. La Edusci..

[27] Kane K.J., Staples K.L. (2014). A group motor skills program for children with coordination difficulties: Effect on fundamental movement skills and physical activity participation. Phys. Occup. Ther. Pediatr..

[28] Ma A.W.W., Fong S.S.M., Guo X., Liu K.P.Y., Fong D.Y.T., Bae Y.H., Yuen L., Cheng Y.T.Y., Tsang W.W.N. (2018). Adapted Taekwondo training for prepubertal children with developmental coordination disorder: A randomized, controlled trial. Sci. Rep..

[29] Balayi E., Sedaghati P. (2021). The effect of combined core stability and coordination exercises on the motor skills of intellectual disability with DCD. Phys. Treat. Specif. Phys. Ther. J..

[30] Hashemi A., Sheikh M., Hemayat-Talab R. (2016). The effect of regular exercise on motor function in children with developmental coordination disorder. Int. J. Sport Stud..

[31] Heus I., Weezenberg D., Severijnen S., Vliet Vlieland T., van der Holst M. (2020). Measuring treatment outcome in children with developmental coordination disorder; responsiveness of six outcome measures. Disabil. Rehabil..

[32] Maharaj S.S., Lallie R. (2016). Does a physiotherapy programme of gross motor training influence motor function and activities of daily living in children presenting with developmental coordination disorder?. S. Afr. J. Physiother..

[33] Zwicker J.G., Rehal H., Sodhi S., Karkling M., Paul A., Hilliard M., Jarus T. (2014). Effectiveness of a summer camp intervention for children with developmental coordination disorder. Phys. Occup. Ther. Pediatr..

[34] Tamplain P., Sherrod G.M., Fuchs C., Miller H.L. (2021). Preliminary improvements in dynamic postural control after a group-based intervention program for children with developmental coordination disorder: A brief report. Dev. Neurorehabilit..

[35] Damanpak S., Sabzi H.A. (2022). The effect of selected motor games on executive functions of children with developmental coordination disorders. Int. J. Pediatr..

[36] Dannenbaum E., Bégin C.L., Daigneault-Bourgeois L., Kwon Pak Yin N., Laferrière-Trudeau C., Mazer B., Moreau V., Salvo L., Villeneuve M., Lamontagne A. (2021). Feasibility and preliminary effects of a 1-week vestibular rehabilitation day camp in children with developmental coordination disorder. Phys. Occup. Ther. Pediatr..

[37] Farhat F., Hsairi I., Baati H., Smits-Engelsman B., Masmoudi K., Mchirgui R., Triki C., Moalla W. (2016). The effect of a motor skills training program in the improvement of practiced and non-practiced tasks performance in children with developmental coordination disorder (DCD). Hum. Mov. Sci..

[38] Fong S.S., Guo X., Cheng Y.T., Liu K.P., Tsang W.W., Yam T.T., Chung L.M., Macfarlane D.J. (2016). A Novel balance training program for children with developmental coordination disorder. Medicine.

[39] Jahanbakhsh H., Sohrabi M., Saberi Kakhki A., Khodashenas E. (2020). The effect of task-specific balance training program in dual-task and single-task conditions on balance performance in children with developmental coordination disorder. Acta Gymnica.

[40] Liu F., Morris M., Hicklen L., Izadi H., Dawes H. (2018). The impact of high and low-intensity exercise in adolescents with movement impairment. PLoS ONE.

[41] Alagesan B., Akahaya J., Rajameena R., Brite Saghaya Rayna A. (2021). Effect of aerobic exercise training on anxiety in children with developmental coordination disorder. Biomedicine.

[42] Mohammadi Oranghi B., Yaali R., Shahrzad N. (2018). The effect of eight weeks aerobic rhythmic exercises with music on motor proficiency, anxiety and depression in children with developmental coordination disorder. Mot. Behav..

[43] Zolghadr H., Sedaghati P., Daneshmandi H. (2019). The effect of selected balance/corrective exercises on the balance performance of mentally-retarded students with developmental coordination disorder. Phys. Treat. Specif. Phys. Ther. J..

[44] Sit C.H., Yu J.J., Wong S.H., Capio C.M., Masters R. (2019). A school-based physical activity intervention for children with developmental coordination disorder: A randomized controlled trial. Res. Dev. Disabil..

[45] Rahman Gholhaki M., Molanorozi K., Ghasemi A. (2021). The effect of selected motor program on neuropsychological variability and motor function at children with developmental coordination disorder. Int. J. Mot. Control Learn..

[46] Miyahara M. (2020). Physical Literacy as a Framework of assessment and intervention for children and youth with developmental coordination disorder: A narrative critical review of conventional practice and proposal for future directions. Int. J. Environ. Res. Public Health.

[47] Monastiridi S., Katartzi E., Kourtessis T., Vlachopoulos S. (2021). A core stabilization program improves motor performance and health-related quality of life in adolescents with motor difficulties. Health Fit. J. Can..

[48] Farhat F., Masmoudi K., Hsairi I., Smits-Engelsman B.C.M., Mchirgui R., Triki C., Moalla W. (2015). The effects of 8 weeks of motor skill training on cardiorespiratory fitness and endurance performance in children with developmental coordination disorder. Appl. Physiol. Nutr. Metab..

[49] Cheng Y.T., Wong T.K., Tsang W.W., Schooling C.M., Fong S.S., Fong D.Y., Gao Y., Chung J.W. (2019). Neuromuscular training for children with developmental coordination disorder. Medicine.

[50] Kordi H., Sohrabi M., Saberi Kakhki A., Attarzadeh Hossini S.R. (2016). The effect of strength training based on process approach intervention on balance of children with developmental coordination disorder. Arc. Argent Pediatr..

[51] Wood G., Miles C.A., Coyles G., Alizadehkhaiyat O., Vine S.J., Vickers J.N., Wilson M.R. (2017). A randomized controlled trial of a group-based gaze training intervention for children with developmental coordination disorder. PLoS ONE.

[52] Noordstar J.J., van der Net J., Voerman L., Helders P.J., Jongmans M.J. (2017). The effect of an integrated perceived competence and motor intervention in children with developmental coordination disorder. Res. Dev. Disabil..

[53] Rezaei S., Arabameri E., Sohrabi M. (2016). Examination of the impact of an eight-week exclusive exercise on the balance of children with developmental coordination disorders. Sci. J. Rehabil. Med..

[54] Norouzi Seyed Hosseini R., Norouzi E., Soleymani M. (2021). Effects of quiet eye training on performance of bimanual coordination in children with DCD. Iran. J. Child Neurol..

[55] Lucas B.R., Elliott E.J., Coggan S., Pinto R.Z., Jirikowic T., McCoy S.W., Latimer J. (2016). Interventions to improve gross motor performance in children with neurodevelopmental disorders: A meta-analysis. BMC Pediatr..

[56] Caçola P.M., Ibana M., Romero M., Chuang J. (2016). The effectiveness of a group motor skill intervention program in children with developmental coordination disorder: Program frequency matters. Internet J. Allied Health Sci. Pract..

[57] Fong S.S., Guo X., Cheng Y.T., Liu K.P., Tsang W.W., Yam T.T., Chung L.M., Macfarlane D.J. (2016). A task-specific balance training improves the sensory organisation of balance control in children with developmental coordination disorder: A randomised controlled trial. Sci. Rep..

[58] Ilana S.D.O., Dayana D.S.O., Julianna D.A.G., Beatriz M.R., Silvia W.S. (2017). Effectiveness of motor intervention on children with Developmental Coordination Disorder (DCD): A systematic review. J. Phys. Educ. Sport Manag..

[59] Yam T.T.T., Or P.P.L., Ma A.W.W., Fong S.S.M., Wong M.S. (2019). Effect of kinesio taping on Y-balance test performance and the associated leg muscle activation patterns in children with developmental coordination disorder: A randomized controlled trial. Gait Posture.

